# Genetic and Physiological Activation of Osmosensitive Gene Expression Mimics Transcriptional Signatures of Pathogen Infection in *C. elegans*

**DOI:** 10.1371/journal.pone.0009010

**Published:** 2010-02-02

**Authors:** Anne-Katrin Rohlfing, Yana Miteva, Sridhar Hannenhalli, Todd Lamitina

**Affiliations:** 1 Department of Physiology, University of Pennsylvania, Philadelphia, Pennsylvania, United States of America; 2 Department of Genetics, Penn Center for Bioinformatics, University of Pennsylvania, Philadelphia, Pennsylvania, United States of America; University of Washington, United States of America

## Abstract

The soil-dwelling nematode *C. elegans* is a powerful system for comparative molecular analyses of environmental stress response mechanisms. Infection of worms with bacterial and fungal pathogens causes the activation of well-characterized innate immune transcriptional programs in pathogen-exposed hypodermal and intestinal tissues. However, the pathophysiological events that drive such transcriptional responses are not understood. Here, we show that infection-activated transcriptional responses are, in large part, recapitulated by either physiological or genetic activation of the osmotic stress response. Microarray profiling of wild type worms exposed to non-lethal hypertonicity identified a suite of genes that were also regulated by infection. Expression profiles of five different osmotic stress resistant (*osr*) mutants under isotonic conditions reiterated the wild type transcriptional response to osmotic stress and also showed substantial similarity to infection-induced gene expression under isotonic conditions. Computational, transgenic, and functional approaches revealed that two GATA transcription factors previously implicated in infection-induced transcriptional responses, *elt-2* and *elt-3*, are also essential for coordinated tissue-specific activation of osmosensitive gene expression and promote survival under osmotically stressful conditions. Together, our data suggest infection and osmotic adaptation share previously unappreciated transcriptional similarities which might be controlled via regulation of tissue-specific GATA transcription factors.

## Introduction

The innate immune system is an evolutionarily ancient response to pathogen infection [1], [2]. The detection of pathogens provokes the upregulation of numerous antimicrobial peptides and cytokines, as well as the activation of more specific adaptive immune mechanisms, via diverse signaling pathways [3]. Molecular mechanisms of innate immunity appear to be highly conserved, since mammals, flies, and worms all utilize similar signaling pathways to activate innate immune responses.

The nematode *C. elegans* has proven to be a powerful model system for genetic and genomic analysis of innate immunity [4]. *C. elegans* can be infected through both the intestine and hypodermis by several bacterial and fungal pathogens, including the human opportunistic pathogen *Pseudomonas aeruginosa* [5]. Unlike infection in humans, infection and killing of *C. elegans* by most bacterial pathogens does not involve cell invasion, replication, and lysis [6]. Several studies have revealed a prominent role for bacterial produced toxins in nematode killing [7], [8], although the pathophysiological mechanisms of such killing in *C. elegans* are still not clear. Nonetheless, genetic screens have led to the identification of a conserved ASK/p38 MAP kinase signaling pathway that is activated by infection and required for proper infection inducible gene expression, toxin defenses, and normal lifespan [8], [9], [10]. While this conserved signaling pathway plays a critical role in immune responses, the upstream sensory mechanisms that activate p38 MAP kinase signaling in response to infection are poorly understood.

In contrast to the clear roles for p38 MAP kinase signaling pathways in the regulation of innate immune responses in *C. elegans*, the transcription factors functioning downstream of p38 MAPKs to execute infection regulated gene expression programs are less well defined. Recent work [11], [12], [13] has suggested that two *C. elegans* GATA-type transcription factors, *elt-2* and *elt-3*, may promote pathogen resistance and pathogen-inducible gene expression in the intestine and hypodermis, respectively. [11], [12], [13]. GATA type transcription factors are zinc finger containing proteins that bind to a highly conserved DNA sequence motif (‘WGATAR’). GATA factors are conserved from yeast to humans and play crucial roles in numerous developmental and physiological contexts. [14]. The specific role(s) of GATA factors in immunity may be evolutionarily conserved since GATAs in both *Drosophila* and humans also play functionally important roles in pathogen resistance [12], [15], [16]. In *Drosophila*, GATAs function together with rel-type transcription factors, such as *Dif*, *Dorsal*, and *Relish* to promote immune responsive gene expression [15]. Rel family transcription factors also mediate responses to a wide variety of cellular stressors [17]. Interestingly, the *C. elegans* genome does not express known homologs of rel-type transcription factors, suggesting that GATA factors may function as key integrators of cellular stressors and immune responses. Currently, it is unclear whether *C. elegans* GATA factors are involved in the response to specific pathogen signals or to more general pathogen-evoked disruptions in host cell homeostatic processes.

In this work, we show that osmotic stress, a natural environmental stress for *C. elegans*, mimics previously described transcriptional responses to bacterial and fungal infection. Transcriptional profiling of five different osmotic stress resistant (*osr*) mutants reiterated the wild type response to osmotic stress and also exhibited a substantial overlap with numerous infection models, suggesting that the overlap is genetically regulated. Using bioinformatics, transgenic promoter analysis, and loss-of-function studies, we found that two GATA factors, *elt-2* and *elt-3*, are required to mediate osmosensitive gene expression in the intestine and hypodermis, respectively. We also find that simultaneous inhibition of both *elt-2* and *elt-3* suppresses physiological and genetic resistance of animals to osmotic stress. These data add to our understanding of the mechanisms of infection by suggesting that osmotic dyshomeostasis may represent a significant pathophysiological consequence of infection in *C. elegans*. Furthermore, our findings further implicate GATA factors as key post-developmental integrators of diverse stress responses in *C. elegans*. Given the highly conserved links between innate immunity and osmoregulation in mammals, understanding the molecular mechanisms of osmosensing and osmosensitive gene expression in *C. elegans* may also shed new light on mechanisms of pathogen-induced cellular toxicity.

## Results

### Microarray Analysis of the Osmotic Stress Response in *C. elegans*

As a soil-dwelling organism, *C. elegans* is exposed to unstable osmotic environments and must adapt to these environments to survive. Previously, we showed that upregulation of a glycerol biosynthetic enzyme resulted in osmotically induced glycerol accumulation, which is required for proper adaptation to hyperosmotic environments [18]. To better understand how these adaptive responses are transcriptionally specified and to define the physiological processes in which osmotic stress responses might be involved, we carried out a time-course microarray analysis of synchronized young adult worms transferred from isotonic (50 mM NaCl Nematode Growth Media(NGM); 170 mOsm) to non-lethal hypertonic (200 mM NaCl NGM; ∼500 mOsm) growth conditions. Labeled cDNA was hybridized to Affymetrix Genechips containing probes against 22,500 transcripts. To capture both acute and chronic responses to hypertonicity, we measured gene expression changes after 15 minutes, 1 hour, 6 hours, and 1 full generation of growth (∼96 hours) on 200 mM NaCl NGM plates. Our previous studies have shown that these levels of osmotic stress result in activation of osmoregulatory adaptive responses, such as the accumulation of the organic osmolyte glycerol and upregulation of the glycerol biosynthetic enzyme *gpdh-1*, without causing significant lethality [18], [19]

**Figure 1:**
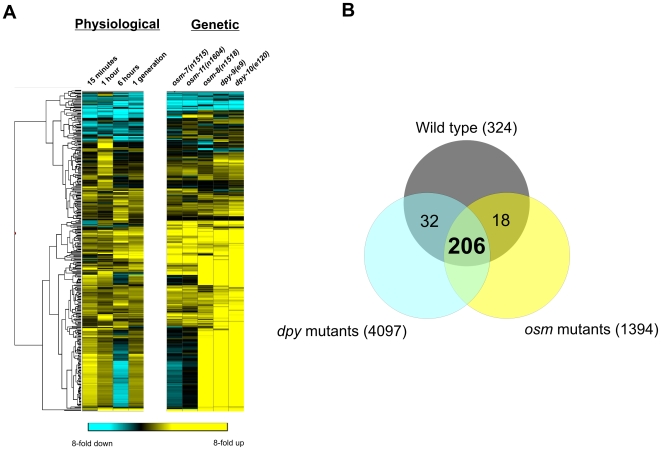
Expression profiling of the hypertonic stress response in *C. elegans*. (A) Hierarchical clustered heat map of gene expression changes associated with physiological activation of osmosensitive gene expression (200 mM NaCl for the indicated time interval) and the regulation of those genes in various osr mutants under normal conditions (50 mM NaCl). For filtering and statistical criteria, see ‘Methods’. (B) Venn diagram showing the overlap between genes differentially regulated by osmotic stress, *osm* mutants, and *dpy* mutants.

Using stringent statistical criteria (see ‘Methods’), we identified 324 genes whose expression was altered ≥3-fold at one or more timepoint (Figure 1A, Table S1) and refer to these as Osmotically *R*egulated *G*enes (ORGs). We validated our microarray data by performing real-time quantitative PCR on a subset of ORGs from independently derived biological samples. In all cases, the qPCR data correlated well with microarray results (Figure S1). Since osmotic stress causes rapid and significant shrinkage of cells due to osmotically induced water loss [19], we considered the possibility that some ORGs might represent a secondary response to osmotically-induced tissue/cellular damage. To separate damage responsive genes from osmotic response genes, we compared the ORG list to genes regulated by the heavy metal cadmium, which has been used previously as a control for the transcriptional effects of tissue damage [8], [12]. Similar to these previous studies [12], ORGs exhibited significant overlap with genes regulated by Cd^2+^ (78/324 genes, 3.1x enrichment, p<0.001). However, of these 78 genes, only 42.3% (33/78) showed concordance in the direction of transcriptional regulation. Furthermore, the makeup of the Cd^2+^ and osmotic stress responses were functionally distinct. For example, Cd^2+^ induced the expression of 14 heat shock proteins, whereas osmotic stress failed to induce any HSPs and repressed the expression of three HSPs. In addition to these differences between ORGs and Cd^2+^ responsive genes, ORGs did not exhibit significant overlap with transcriptional responses to either ER stress or hypoxia, two additional stressors that are also thought to cause tissue damage [20], [21]. (Figure S2). Together, these data show that the majority of the transcriptional response to osmotic stress is largely distinct in both composition and function from other damage-inducing environmental stressors.

### OSR Mutants Activate the ORG Transcriptional Program in the Absence of Osmotic Stress

To further determine whether activation of ORGs was responsive to tissue damage or to genetically regulated activation of osmosensitive signaling pathways, we performed microarray analysis of five osmotic stress resistant (*osr*) mutants grown under isotonic conditions. *osr* mutants exhibit many of the physiological characteristic of wild type animals adapted to hyperosmotic stress, such as high *gpdh-1* expression, glycerol accumulation, and resistance to normally lethal levels of hypertonicity [18], [22]. However, unlike wild type animals, *osr* mutants mis-express these phenotypes under isotonic conditions, suggesting that *osr* mutants encode genes that are negative regulators of osmosensitive signaling pathways and that loss of function mutations in *osr* genes cause constitutive activation of these pathways. The *osr* mutants *osm-7* and *osm-11* encode homologous secreted proteins that may also function as Notch ligands [22], [23], *dpy-9* and *dpy-10* encode extracellular matrix collagens, and *osm-8* encodes a secreted mucin-like protein (Rohlfing and Lamitina, in preparation). *osr* mutants exhibit no obvious signs of cellular or tissue damage under isotonic conditions [22] (our unpublished observations). Therefore, the genes transcriptionally regulated by *osr* mutants are likely responsive to osmosensitive signaling pathways rather than secondarily responsive to tissue damage.

Using stringent statistical criteria (see ‘Methods’), we found that the genes regulated by *osr* mutants are largely overlapping with each other and with wild type ORGs (Figure 1B, Tables S2, S3, S4, S5, S6). Activation or repression of gene expression tended to be more robust in the *osr* mutants when compared to wild type animals exposed to 200 mM NaCl (Figure 1A), suggesting that *osr* mutants cause strong and constitutive osmoresponsive gene expression. Of the genes physiologically regulated by osmotic stress in wild type animals, 63.5% (206/324) were also regulated by all five *osr* mutants under isotonic conditions (Figure 1B), which is a highly significant enrichment (p<0.001). The transcriptional profiles of the five *osr* mutants fell into two distinct patterns, with the Notch ligands mutants *osm-7* and *osm-11* and the mucin protein mutant *osm-8* exhibiting a high degree of similarity to the wild type ORG dataset and the collagen *dpy-9*, and *dpy-10* regulating a partially overlapping and much larger set of transcripts that was less similar to the wild type ORG dataset (Figure S3). Genes specifically regulated by the *osm-7* and *osm-11* mutants were not enriched for known Notch signaling targets [24], suggesting that these proteins may have other regulatory roles in addition to their roles in Notch signaling. *osr* mutant transcriptional profiles did not exhibit significant overlap with transcriptional responses to heat shock, hypoxia, or ER stress, further demonstrating that *osr* mutant activation of osmosensitive gene expression is highly specific (data not shown). Taken together, these data suggest that the *osr* mutants genetically activate a transcriptional program that is also physiologically activated by hyperosmotic stress in wild type *C. elegans*.

### OSR Mutants and Osmotic Stress Mimic Innate Immune Transcriptional Responses

**Figure 2:**
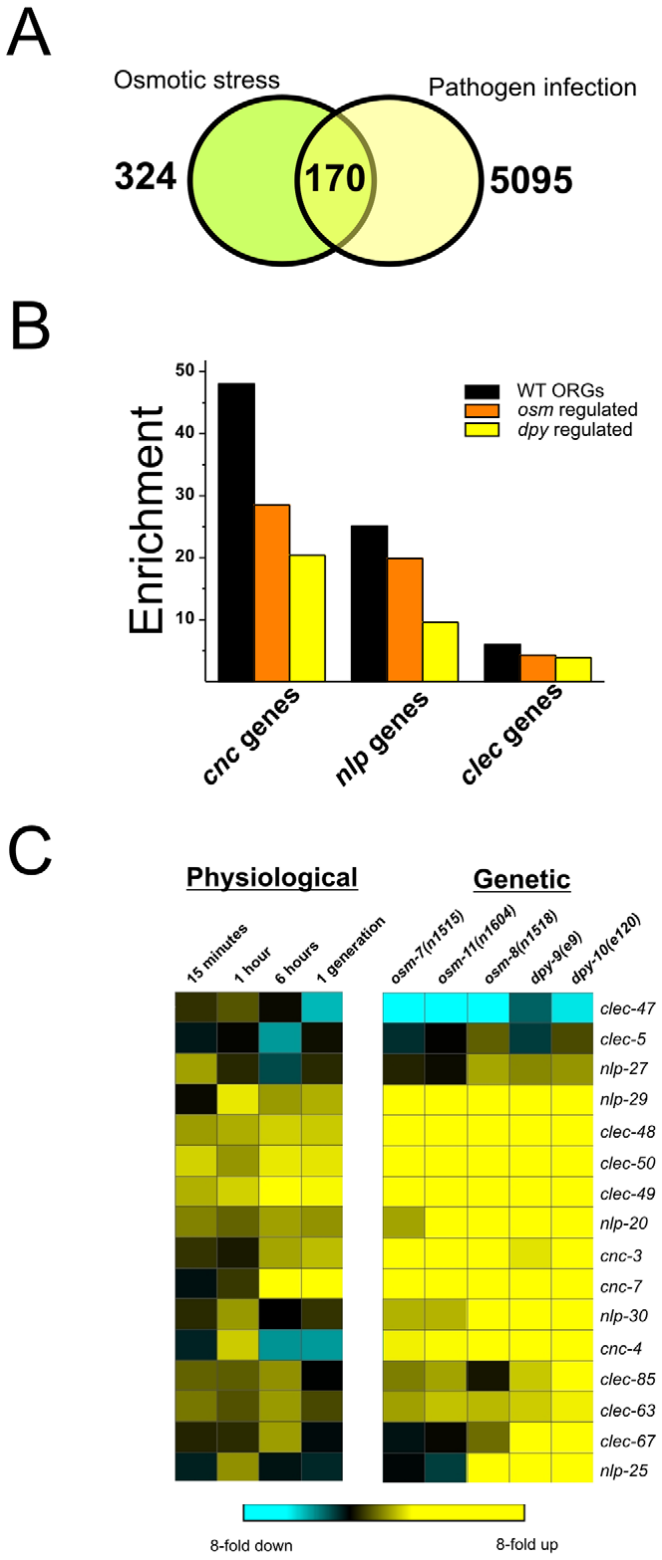
Osmotically regulated genes exhibit significant overlap with pathogen regulated genes. (A) Venn diagram showing the genes in common between osmotic stress and a compilation of all pathogen microarray data. Pathogen data were compiled from existing microarray datasets [10], [12], [25], [26] to obtain 5095 unique genes that were significantly regulated in at least one model of infection. (B) Enrichment of the indicated gene class among genes regulated by osmotic stress in wild type or under isotonic conditions in *osm* and *dpy* mutants. (C) Hierarchical clustering of antimicrobial *cnc*, *nlp*, and *clec* gene expression in osmotically stressed wild type or unstressed *osm* and *dpy* mutants.

Among the genes regulated by both osmotic stress and the *osr* mutants, we noticed an enrichment for several genes and gene classes previously associated with microbial infection in *C. elegans*. These included neuropeptide like proteins (*nlp*), caenicin (*cnc*), and c-type lectin (*clec*) gene classes (Figure 2B). These genes are thought to function as antimicrobial peptides in *C. elegans* and many are induced by both bacterial and fungal infection [11], [25]. We found that virtually all of these immune effectors were strongly upregulated by both osmotic stress and *osr* mutants (Figure 2C). To test whether ORGs overlapped more broadly with infection regulated genes, we compiled a list of all genes known to be regulated by bacterial or fungal infection in *C. elegans* and compared this list with our ORG dataset [10], [11], [12], [25], [26]. We found that 52.4% of ORGs (170/324) were differentially regulated in at least one model of infection, which represented a significant enrichment over that expected for two randomly distributed datasets (1.4 fold enrichment, p<0.001; Figure 2A). ORGs exhibited significant overlap with infection regulated genes from 6/8 datasets representing exposure to seven different pathogen models (Table S7). Among all pathogen datasets, ORGs exhibited the most significant enrichment for genes regulated by infection with the gram positive pathogen *M. nematophilium*, which induces a protective inflammatory cell swelling response in the post-rectal epithelial cells [27]. ORGs also exhibited a highly significant enrichment in genes regulated by *P. aeuroginosa*, whose mechanism of killing is strongly influenced by environmental osmolarity [7]. While the expression of many ORGs and infection regulated genes, such as the *nlp*, *cnc*, and *clec* genes, exhibited concordance in their regulation under osmotic stress and infection conditions, some genes, such as the organic osmolyte accumulation enzyme *gpdh-1*, which encodes a glycerol-3-phosphate dehydrogenase, was strongly upregulated by osmotic stress but downregulated by most models of infection. These data show that genes transcriptionally regulated by both osmotic stress and infection are highly similar in many respects. However, *C. elegans* can discriminate osmotic stress from infection, as evidenced by the specific upregulation of *gpdh-1* by hyperosmotic stress.

### The Promoters of ORGs Are Enriched for GATA Transcription Factor Binding Motifs

**Figure 3:**
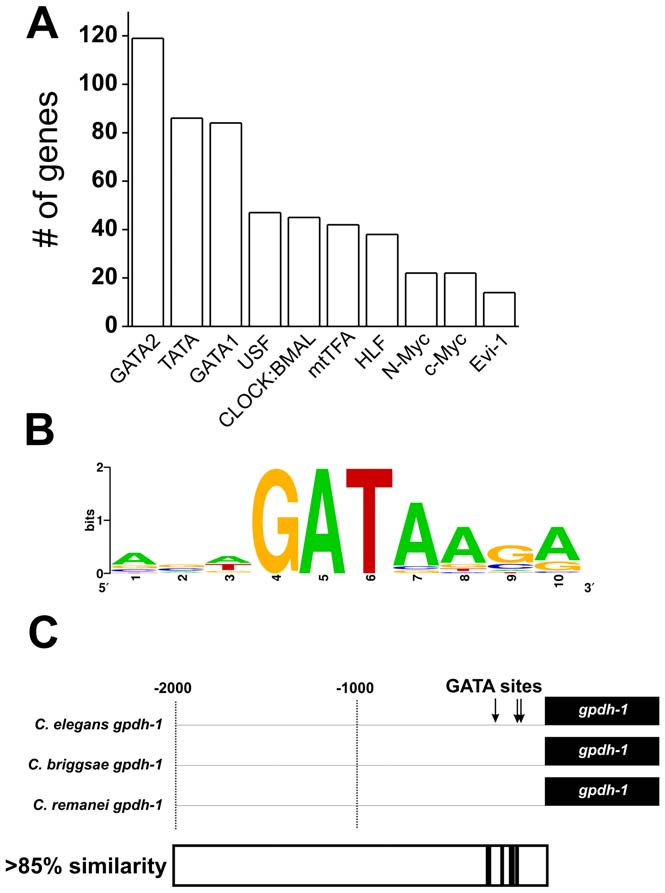
GATA transcription factor binding sites are over-represented among upregulated ORGs. (A) TRANSFAC-annotated transcription factor DNA binding motifs with enrichment scores >1.5 and p-values<0.05 in both *C. elegans* and *C. briggsae* ORGs. The number of ORGs with each motif is plotted. For details of enrichment analysis, see ‘Methods’. Only binding motifs with p-values of <0.05 in both *C. elegans* and *C. briggsae* datasets are shown. GATA - GATA binding protein 2; TATA - TATA sequence binding protein; USF - Upstream stimulatory factor (USF1 and USF2); Clock:BMAL - brain-muscle-ARNT-like protein 2 (ARNT-2); mtTFA - mitochondrial transcription factor A; HLF - hepatic leukemia factor; N-Myc - neuroblastoma MYC oncogene; c-Myc - c-myc proto-oncogene; Evi-1 - Ecotropic viral integration site 1 transcription factor. (B) Consensus GATA2 motif from 181 occurrences in the *C. elegans* ORG dataset. (C) Analysis of the gpdh-1 promoter from *C. elegans*, *C. briggsae*, and *C. remanei*. Regions containing >85% identify among all three promoters are indicated in black shading.

We used single gene RNA interference to test the *in vivo* functional requirements for 280/324 ORGs but failed to observed significant affects on survival, growth, and reproductive capacity in osmotically challenged wild type animals or in various sensitized genetic backgrounds (*rrf-3*, *gpdh-1*, *gpdh-2*, *gpdh-1;gpdh-2*, and *osm-11;* data not shown). This suggested that, like other complex physiological processes such as ageing [28], the transcriptional responses of individual genes play subtle roles in the response to hyperosmotic stress. Given these results, we hypothesized that regulatory genes, such as transcription factors, might exhibit more robust phenotypes with regards to the activation of osmosensitive gene expression and *in vivo* osmotic stress resistance. To identify transcription factors that might regulate osmotic stress responses *in vivo*, we first performed an unbiased search of the promoters (1Kb) of all upregulated ORGs and their *C. briggsae* orthologs for transcription factor binding sites using all binding motifs described in the TRANSFAC motif database (www.gene-regulation.com; see ‘Methods’). Using this approach, we identified several transcription factor binding motifs that were enriched in the ORG dataset (Figure 3A). The broadest and most statistically significant motif enrichment was for the GATA type transcription factor binding motif (Figure 3A). The consensus GATA motif found in the promoters of ORGs is highly similar to the previously published motif for *C. elegans elt-2* (Figure 3B; ‘ACTGATAAGG’) [29]. Within the promoter of the most strongly regulated ORG *gpdh-1*, GATA sites were located ∼250 bp upstream of the ATG start codon. This region shows high sequence conservation between multiple nematode species (Figure 3C), suggesting that GATA sites and/or additional motifs might play functionally important roles in the activation of osmosensitive *gpdh-1* expression.

### GATA Sites Are Required for Physiological and Genetic Regulation of *gpdh-1* Expression in the Intestine and Hypodermis

**Figure 4:**
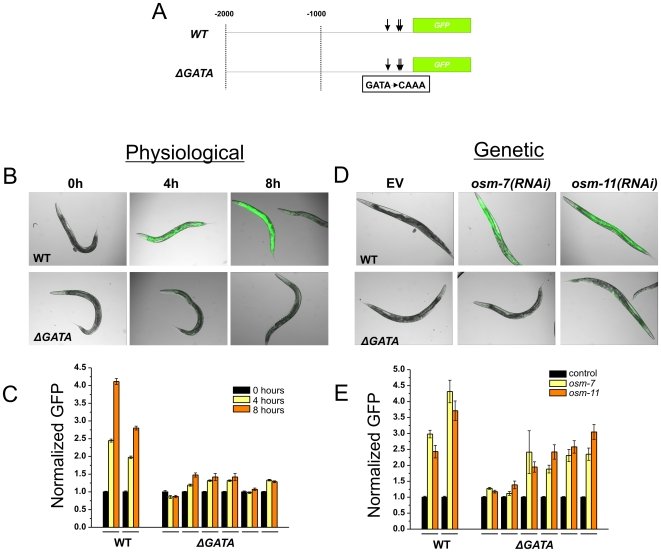
GATA binding sites are required in *cis* for physiological and genetic activation of *gpdh-1* expression. (A) Schematic diagram of the *gpdh-1* promoter constructs, with the location of GATA binding sites and the mutagenized sequences indicated by arrows. (B) Animals expressing either the wild type (top) or ΔGATA *gpdh-1p::GFP* transgene (bottom) were exposed to 200 mM NaCl for 4 or 8 hours before being imaged (B) or quantified using the COPAS Biosort (C). For COPAS data, n>100 young adult animals for two independent wild type lines and six independent ΔGATA lines. (D) Animals carrying either the wild type (top) or the ΔGATA *gpdh-1p::GFP* transgene (bottom) were fed *osm-7(RNAi)* or *osm-11(RNAi)* for two generations. The RNAi treatment was highly effective in all lines, as 100% of *osm-7(RNAi)* or *osm-11(RNAi)* animals exhibited an osmotic stress resistance (*osr*) phenotype. (E) The induction of GFP following EV, *osm-7*, or *osm-11* RNAi was measured on the COPAS Biosort; n>100 for all lines. Horizontal lines indicate data from independent transgenic lines.

To determine whether GATA binding sequences were required for genetic and physiological activation of *gpdh-1* expression, we constructed a series of *gpdh-1* promoter deletions joined to the coding sequence for GFP and analyzed their ability to mediate osmosensitive GFP expression in live transgenic animals. Constructs containing 251 bp base pairs of promoter sequence, which contain all conserved GATA binding sites, were sufficient to mediate osmosensitive GFP expression (Figure S4). However, constructs containing 114 bp of promoter sequence, which did not include consensus GATA sites, failed to induce GFP when exposed to hypertonic conditions, suggesting that sequences within the −114 to −250 region are required for activation of osmosensitive *gpdh-1* expression. Since this region contains three predicted GATA binding sites, we hypothesized that these sites were required in *cis* to mediate osmosensitive *gpdh-1* expression. To test this hypothesis, we utilized site directed mutagenesis to convert the consensus ‘GATA’ sequence to ‘CAAA’ in the context of a full length (3.0 Kb) *gpdh-1* promoter for all three predicted GATA binding sites (Figure 4A). Similar alterations have been shown to inhibit GATA-DNA interactions and block transcriptional transactivation [30]. Transgenic animals expressing either wild type or mutated GATA constructs were analyzed for their ability to respond to both physiological activation by high salt (Figure 4B,C) and genetic activation by *osr* mutants (Figure 4D,E). The wild type *gpdh-1p::GFP* expression was robustly induced in the hypodermis and intestine of wild type animals following brief (4 h or 8 h) exposure to 200 mM NaCl (Figure 4B,C). RNAi inactivation of both *osm-7* and *osm-11*, which negatively regulate *gpdh-1* expression [18], also resulted in robust GFP expression in the intestine and hypodermis under isotonic conditions (Figure 4D,E). In contrast, all ΔGATA-*gpdh-1p::GFP* transgenic lines failed to express significant levels of GFP even after 8 hours of exposure to 200 mM NaCl (Figure 4B,C). Genetic activation of *ΔGATA*-*gpdh-1p::GFP* by inhibition of *osm-7* and *osm-11* was also strongly attenuated. Interestingly, in response to genetic activation, intestinal expression was completely eliminated in *ΔGATA*-*gpdh-1p::GFP*, but hypodermal expression frequently persisted in both mutants (Figure 4D,E), suggesting that additional factors are likely to participate in the regulation of *gpdh-1* expression in the hypodermis. These data demonstrate that GATA motifs are absolutely required in *cis* for robust physiological and genetic activation of *gpdh-1p::GFP* expression in the intestine and partially required for genetic activation of *gpdh-1p::GFP* in the hypodermis.

### Tissue-Specific GATA Factors *elt-2* and *elt-3* Function Together to Mediate *In Vivo* Osmosensitive Gene Expression and Whole Animal Osmotic Stress Resistance

**Figure 5:**
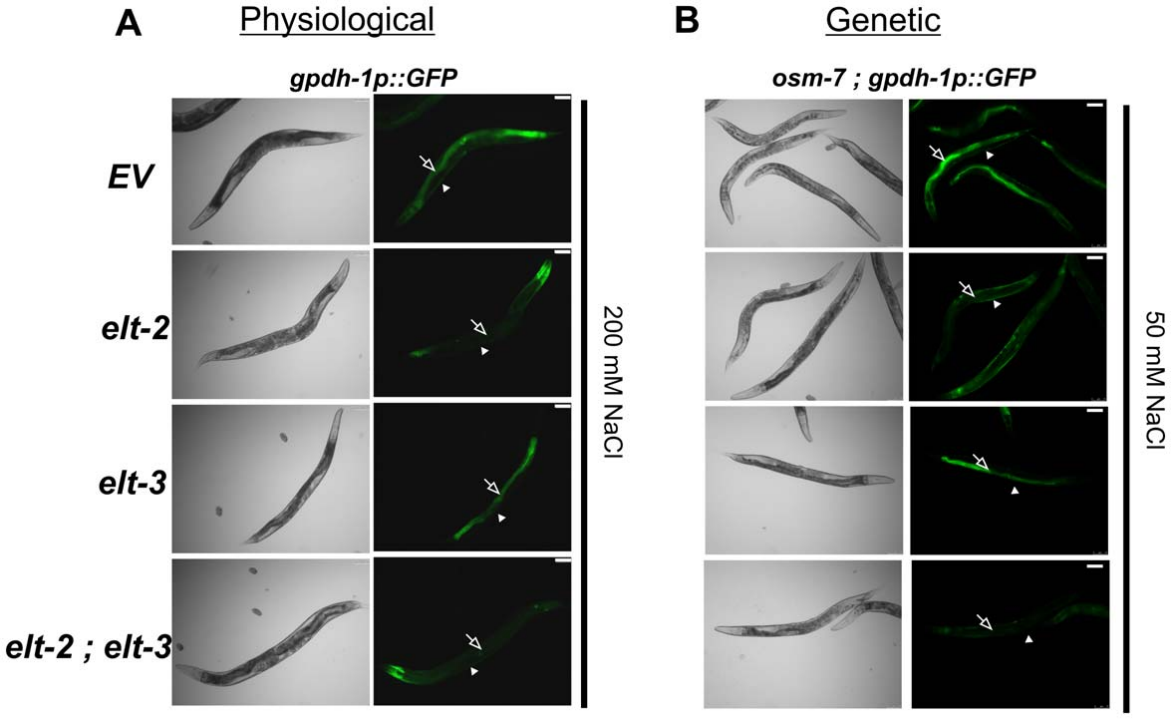
The GATA factors *elt-2* and *elt-3* mediate cell-type specific activation of *gpdh-1* expression following physiological or genetic activation. (A) *gpdh-1p::GFP* animals exposed to the indicated RNAi treatments were exposed to 200 mM NaCl for 24 hours and GFP was imaged. Scale bar = 75 µ. (B) *osm-7(n1515); gpdh-1p::GFP* animals were exposed to the indicated RNAi treatments under isotonic conditions and GFP was imaged. Scale bar = 75 µ. Open arrows point to the intestine and closed arrowheads point to the hypodermis.

The above data suggested that one or more GATA factors may play functionally important roles in the regulation of osmosensitive gene expression and *in vivo* osmotic stress resistance in *C. elegans*. Previous studies demonstrated that the GATA factor *elt-3* is necessary for osmotically induced gene expression in the hypodermis [11] and we confirmed this result (Figure 5). To determine whether additional GATA factors regulate intestinal *gpdh-1* expression, we used RNAi to post-embryonically inhibit the expression of 10/14 *C. elegans* GATA factors in animals expressing *gpdh-1p::GFP*. We found that RNAi against one GATA factor, *elt-2*, attenuated osmotically induced GFP expression in the intestine without substantially affecting hypodermal GFP expression (Figure 5). *elt-2(RNAi)* also suppressed the genetic activation of intestinal, but not hypodermal, *gpdh-1p::GFP* expression in the *osr* mutant *osm-7* (Figure 5). The effect of *elt-2(RNAi)* on osmotic stress responses was not simply due to a general defect in intestinal gene expression, since *elt-2(RNAi)* exhibited normal expression of an intestinal ER-stress reporter (*hsp-4p::GFP*) (data not shown).

**Figure 6:**
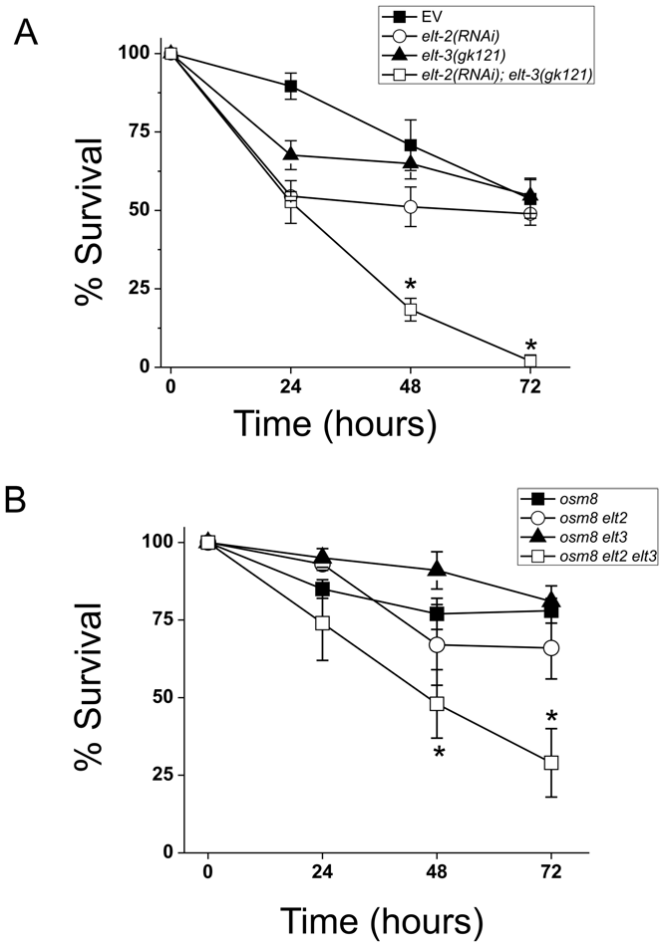
The GATA factors *elt-2* and *elt-3* are required to mediate physiological and genetic resistance to hyperosmotic stress in *vivo*. (A) Wild type or *elt-3(gk121)* animals were exposed to either control (EV) or *elt-2(RNAi)* treatment as described in ‘Methods’. Young adult animals were transferred to 500 mM NaCl plates and survival was measured each 24 hour period. Animals that died from bagging or crawling up the edge of the plate were censored at the time of the event. N>50 animals per genotype. (B) *osm-8(n1518)* or *osm-8(n1518); elt-3(gk121)* animals were exposed to either control (EV) or *elt-2(RNAi)* treatment as described in ‘Methods’. Young adult animals were transferred to 500 mM NaCl and survival was measured each 24 hour period. Animals that died from bagging or crawling up the edge of the plate were censored at the time of the event. N>40 animals per genotype. * - p<0.05.

Previous studies have shown that both *elt-3* and *elt-2* play functionally important roles in the innate immune responses to both fungal and bacterial pathogens [11], [12], [13]. Since osmotic stress induces robust transcriptional responses in both the hypodermis and intestine, we hypothesized that hypodermal *elt-3* and intestinal *elt-2* might play additive roles in mediating *in vivo* osmotic stress resistance. To test this hypothesis, we measured the survival of *elt-2(RNAi)*, *elt-3(gk121)*, and double mutant *elt-2(RNAi); elt-3(gk121)* animals following exposure to hyperosmotic stress. Each of the single *elt* mutants survived a hyperosmotic challenge as well as wild type animals (Figure 6A). However, the *elt-2; elt-3* double mutant showed greater sensitivity to hyperosmotic stress that either of the single mutants (Figure 6A). We also tested whether *elt-2* and/or *elt-3* were required for genetically endowed osmotic stress resistance using the *osr* mutant *osm-8*. The chronic osmotic stress resistance phenotype of *osm-8(n1518)* mutants was not significantly suppressed in either *elt-2(RNAi)* or *elt-3(gk121)* backgrounds (Figure 6B). However, *osm-8(n1604); elt-2(RNAi); elt-3(gk121)* triple mutants exhibited a significant reduction in chronic osmotic stress resistance (Figure 6B). The phenotype of the triple mutants was not due to defects in the general health of the animals, since survival under isotonic conditions was indistinguishable from control animals (data not shown). These data show that the GATA factors *elt-2* and *elt-3* play overlapping and/or redundant roles in both physiological and *osm-8*-induced genetic regulation of in *vivo* osmotic stress resistance in *C. elegans*.

## Discussion

Previous studies of infection in *C. elegans* have noted that transcriptional responses to infection appear highly specific and exhibit little significant overlap with other forms of well-studied environmental stress, such as heat shock and ER stress [12], [13]. However, infection by diverse types of pathogens gives rise to common transcriptional signatures [26], the etiology of which is not clear. Here, we find that activation of the osmotic stress response in *C. elegans*, either through physiological or genetic mechanisms, can mimic common transcriptional responses to a diverse array of infection models in *C. elegans*. The mechanism(s) by which activation of osmotic stress responses mimic infection is currently unclear but one intriguing mechanism may relate to infection-induced disruptions in osmotic homeostasis. For example, many pathogens, via their production of pore-forming toxins and Type III secretion systems, create ion and water permeable pores in the plasma membrane [31]. Due to the standing electrochemical ion gradients present in all cells, the insertion of these relatively non-selective pores will allow such gradients to collapse, producing osmotic dyshomeostasis [32], [33]. In this regard, osmotic stress may mimic early pathophysiological responses to infection. Consistent with this hypothesis, there is also a substantial overlap between the ORGs identified in this study and genes regulated by exposure of *C. elegans* to a purified pore-forming toxin [8]. In the future, it would be interesting to test whether common transcriptional signatures of infection in *C. elegans* are reduced following infection with toxin-compromised pathogens.

Functional links between infection and osmotic stress have also been observed in other systems. For example, in mammals, T-cell activation in the thymus occurs in hypertonic environments, the maintenance of which requires the rel-type transcription factor NFAT5/TonEBP [34]. NFAT5/TonEBP is also an essential mediator of hypertonicity-induced transcriptional responses in diverse cell types, including the extremely hypertonic environment of the inner medulla of the mammalian kidney [35], [36]. In *Drosophila*, salt stress induces a broad transcriptional response that includes the induction of numerous infection regulated genes [37]. Along with the present studies in *C. elegans*, these data suggest the intriguing hypothesis that cellular osmotic stress may be an evolutionarily conserved and physiologically significant activator of immune responses. Interestingly, the *C. elegans* genome does not contain rel-type transcription factors similar to NFAT5/TonEBP, suggesting that the functional links between osmotic stress and immunity may have preceded the evolution of this family of transcription factors. *C. elegans* should provide an outstanding model system in which to better explore the functional, molecular, and evolutionary relationships between immunity and osmotic stress.

In animals, the molecular mechanisms regulating osmosensing and osmosensitive gene expression are not well understood. As mentioned above, the rel factor NFAT5 is essential for osmosensitive gene expression in mammals, yet *C. elegans* does not express a homolog of this or any other rel factor. Therefore, the transcription factor(s) controlling osmosensitive gene expression in *C. elegans* have remained mysterious. Our data reveal an important role for two GATA-type transcription factors, *elt-2* and *elt-3*, in this process. Both GATA factors are also required for other developmental and non-developmental processes [11], [12], [13], [38], [39], [40], [41], [42], suggesting that additional factors most likely coordinate with *elt-2* and *elt-3* to determine their specific responses. For example, while *elt-2* is required for both osmosensitive and infection-inducible gene expression, the *sek-1*/*pmk-1* p38 MAP kinase signaling pathway is only required for pathogen-induced transcriptional responses and not osmosensitive gene expression [11] (our unpublished observations). Therefore, multiple upstream signaling pathways may converge on GATA factors to determine their specific transcriptional outputs. Such convergence may lead to an enrichment of GATA factors at signaling-appropriate promoters, possibly through interactions with specific transcriptional cofactors or other signaling components. Such a mechanism could explain how inducible activation of GATA factors might regulate a more restrictive set of transcriptional targets. It is also intriguing to note the parallels between our findings on the role of *elt-2* and studies of another developmentally required transcription factor, *skn-1*, which is required for oxidative stress responses in the *C. elegans* intestine. Multiple signaling pathways converge on *skn-1*, which integrates these signals to coordinate appropriate responses to ageing, insulin signaling, caloric restriction, and oxidative stressors [43], [44], [45], [46], [47]. Further studies will be required to determine whether *elt-2*, like *skn-1*, exhibits direct regulation of localization, DNA binding, and/or activity in response to osmotic stress and/or infection.

In conclusion, our findings show that both osmotic stress and pathogen infection regulate similar transcriptional targets in *C. elegans*. Tissue-specific GATA-type transcription factors *elt-2* and *elt-3* play a major role in coordinating both of these gene expression programs and are required for the response to osmotic stress. Our studies functionally link cellular osmotic stress responses with innate immunity and suggest that osmotic dyshomeostasis might represent a new and evolutionarily significant paradigm for the physiological basis of host cell pathogen sensing.

## Materials and Methods

### *C. elegans* Strains and Transgenics

All strains were maintained using standard culture methods and fed with the *E. coli* strain OP50. NGM media was made hypertonic by the addition of NaCl to the indicated concentration. The following strains were used: LGI: *kbIs5(gpdh-1p::GFP; rol-6);* LGII: *dpy-10(e128)*, *osm-8(n1518)*; LGIII: *osm-7(n1515)*; LGIV: *dpy-9(e12)*; LGX: *osm-11(n1604)*, *elt-3(gk121)*. Unless otherwise noted, all strains were grown at 20°C. The wild type N2 strain was obtained from the *Caenorhabditis elegans* Genetic Stock Center.

*gpdh-1::GFP* reporter constructs were created using PCR or site directed mutagenesis of the construct described in [18]. Site directed mutagenesis was carried out using the QuickChange System (Stratagene) and all mutations were verified by DNA sequencing. For both deletion and ΔGATA transgenics, wild type animals were injected with 150 ng/µl of a *myo3p::dsRed2* plasmid and 30 ng/µl of the relevant *gpdh-1* construct using standard microinjection methods [48]. For quantification, animals carrying the extrachromosomal arrays were placed into a COPAS Biosort (Union Biometrica) and animals expressing dsRed were separated from non-transgenic animals in the gating window. GFP expression from dsRed+ animals was normalized to worm size (time-of-flight) and each sample was normalized to the mean of the corresponding control, as indicated in each figure.

### *C. elegans* Growth and Collection for Microarray Analysis

Hypochlorite synchronized L1 stage wild type animals were grown to the young adult stage on standard NGM plates. Worm were washed off plates using isotonic M9 solution (∼170 mOsm) and placed onto NGM plates containing 200 mM NaCl for 15 minutes, 1 hour, or 6 hours. For the steady state samples, synchronized L1 stage animals were placed onto NGM plates containing 200 mM NaCl and allowed to grow to the young adult stage. For all samples, 1000 adult worms were collected into 1.5 ml tubes using gating criteria on the COPAS Biosort that excluded L4 and younger animals. Immediately following sorting, worms were pelleted at 2,000 RPM and the supernatant was aspirated, leaving ∼100 µl on top of the worm pellet. 400 µl of Trizol was added and the solution was vortexed for two minutes. Worms were stored at -80° until RNA isolation. For each timepoint, five individual replicates were performed. The best three preparations (as determined by Agilent Bioanalyzer analysis and spectrophotometric readings) were used for RNA labeling and hybridization. The remaining two preparations were used for qPCR validation of microarray results.

### RNA Isolation and Microarray Analysis

Total RNA was extracted using a combined Trizol (Invitrogen)/RNAeasy (Qiagen, Valencia, CA) column method. Purified total RNA was submitted to the University of Pennsylvania Microarray Core facility for RNA quality control, labeling, and hybridization to Affymetrix *C. elegans* GeneChips. All protocols were conducted as described in the Affymetrix GeneChip Expression Analysis Technical Manual. Briefly, 2 µg of total RNA was converted to first-strand cDNA using Superscript II reverse transcriptase primed by a poly(T) oligomer that incorporated the T7 promoter. Second-strand cDNA synthesis was followed by in vitro transcription for linear amplification of each transcript and incorporation of biotinylated CTP and UTP. The cRNA products were fragmented to 200 nucleotides or less, heated at 99°C for 5 min and hybridized for 16 h at 45°C. The microarrays were then washed at low (6X SSPE) and high (100 mM MES, 0.1M NaCl) stringency and stained with streptavidin-phycoerythrin. Fluorescence was amplified by adding biotinylated anti-streptavidin and an additional aliquot of streptavidin-phycoerythrin stain. A confocal scanner was used to collect fluorescence signal at 3 um resolution after excitation at 570 nm. The average signal from two sequential scans was calculated for each microarray feature.

Affymetrix .cel files for all arrays were uploaded into the Partek Genomics Suite and intensity values were normalized using the GC-RMA algorithm. Using Affymetrix present/absent calls, 13,866/22,625 probes were ‘present’ in at least 3/30 datasets, and these probes were used for all subsequent statistical analysis. Data across the wild type series was analyzed using the Significance analysis of Microarrays (SAM) algorithm (to calculate the False Discovery Rate (FDR)[49]) and genes with a ≤1% FDR and ≥3-fold change were considered Osmotic Regulated Genes (ORGs). Data were further manually curated to add annotation or remove pseudogenes or genes no longer predicted to be expressed using data available in Wormbase (WS190), resulting in the final list of 324 ORGs. All microarray data are MIAME compliant and have been deposited in the Gene Expression Omnibus (GEO) database under accession number GSE19310.

### Quantitative PCR

cDNA was reverse synthesized from 1 µg total RNA (Superscript II kit, Invitrogen, Carlsbad, CA) and qPCR was performed using an ABI7300 thermocycler and TaqMan probe sets (Applied Biosystems, Foster City, CA). Each reaction was performed in 20 µl reactions in technical triplicate or quadruplicate from 2–3 biological replicates. Data were normalized to expression levels of *cpt-6*, which encodes a carnitine palmitoyl transferase whose expression levels are unchanged by hypertonicity. Initial test showed that data normalization using probes against *clh-3* and *lam-3*, whose expression was also unchanged by hypertonicity, showed similar results to those achieved with *cpt-6* (data not shown).

### Survival Assays, RNA Interference, and COPAS Biosort Analysis

Survival assay were performed by placing 10–20 young adult animals onto OP50-seeded NGM plates containing the indicated amount of NaCl. Animals were scored for survival after 24 hours. Worms were considered dead when they failed to respond to harsh plate tapping and light touch with a platinum wire. Animals that died as the result of internally hatched progeny (“bag-of-worms” phenotype) were censored from the analysis. Each NaCl concentration was repeated in quadruplicate and each assay was repeated 2–3 times. Figures illustrate the data from one representative experiment.

For the *elt-2* RNAi experiments, L1 stage animals were fed control RNAi bacteria (HT115 containing the pPD129.36 empty vector (EV) plasmid) for 24 hours at 16°, washed from plates, and distributed to EV or *elt-2(RNAi)*(from MRC RNAi library; sequence confirmed) plates for 72 hours at 16° prior to either survival assays or COPAS measurements. Using these conditions, we were able to circumvent the developmental arrest phenotype of *elt-2(RNAi)* and still achieve ∼90% knockdown of *elt-2* mRNA, as assessed by qPCR (data not shown). For survival assays, *EV(RNAi)* and *elt-2(RNAi)* animals were transferred to NGM RNAi plates containing the indicated levels of NaCl and survival was measured as described above. For COPAS assays, *kbIs5* animals were washed onto new RNAi plates containing the indicated RNAi clone and either 50 mM or 200 mM NaCl. At the indicated timepoints, animals were analyzed on a COPAS Biosort for time-of-flight (TOF) and GFP expression. Raw data were filtered to select adult animals (TOF>300) and the GFP data was normalized to the TOF measurement (GFP/TOF).

### Motif Analysis

1 kb of promoter sequence (upstream of the start ATG) from each gene in Groups 2–5 of the *C. elegans* ORG dataset (foreground dataset) and their *C. briggsae* orthologs were searched for the presence of a transcription factor binding motif using the 811 motifs or Positional weight Matrices (PWM) in TRANSFAC 10.2 (www.gene-regulaiton.com). We also performed the search using 8451/8956 *C. elegans/C. briggsae* ortholog pairs that exhibited detectable expression in our microarray data (background dataset) The motif scan was done using a previously described tool, PWM_SCAN [50]. A stringent percentile score threshold of 0.95 was used to consider a motif “present” in a gene promoter. The percentile score of 0.95 means that the score was above the 95^th^ percentile of range of scores achievable by a PWM. For a motif, given the number of matches in the foreground dataset (ORGs), the p-value corresponding to the motif occurrence was estimated based on 1000 random samplings of the background dataset and computing the fraction of times that the motif occurence equaled or exceeded its occurrences in the foreground dataset. Only motifs with p-values<0.05 in both the *C. elegans* and *C. briggsae* datasets were considered significant.

### Statistical Analysis

Data are presented as means ± S.E. Survival and qPCR gene expression data were analyzed using either the Student's T-test or one-way ANOVA, as implemented in Graphpad Prism (San Diego, CA). Enrichment scores and enrichment significance were calculated as previously described [51]. For enrichment analysis, we used 13,866 (the number of expressed genes in our ORG microarrays) as the total number of genes. P-values<0.05 were taken to indicate statistical significance.

## Supporting Information

Figure S1 qPCR validation of hypertonic stress microarray data. cDNA prepared from two independent biological replicates was used as the template for quantitative RT-PCR (see methods). Data shown are the mean fold changes (hypertonic/isotonic) ± S.D. Microarray data are the mean fold changes from 3 independent samples.
(0.47 MB TIF)

Figure S2 ORGs do not exhibit significant overlap with other stress-induced transcriptional responses. ORGs were compared to previously published microarray studies of the endoplasmic reticulum unfolded protein response (UPR) and the response to hypoxia [20], [21]. The enrichment score and the hypergeometric p-value of enrichment was calculated as described previously [49].
(0.38 MB TIF)

Figure S3 Pearson Correlation analysis between WT ORGs and osr mutants. The pairwise Pearson Correlation coefficient was determined between each dataset. Each experimental replicate is shown under the indicated genotype.
(0.77 MB TIF)

Figure S4 Truncation analysis of the gpdh-1 promoter. Transgenic animals containing the indicated lengths of the gpdh-1 promoter (from start ATG) were fused to GFP using a PCR fusion method [52]. GFP expression in transgenic animals was quantified on a COPAS Biosort following exposure to either 50 mM NaCl or 200 mM NaCl. N>150 animals for each measurement.
(0.42 MB TIF)

Table S1 Osmotically regulated genes in wild type following 15 minute, 1 hour, 6 hour, and 1 generation of exposure to 200 mM NaCl and their regulation in five *osr* mutants (Excel file).
(0.10 MB XLS)

Table S2 Genes regulated in *osm-7(n1515)* mutants and their regulation in four other *osr* mutants (Excel file).
(0.06 MB XLS)

Table S3 Genes regulated in *osm-8(n1518)* mutants and their regulation in four other *osr* mutants (Excel file).
(0.23 MB XLS)

Table S4 Genes regulated in *osm-11(n1604)* mutants and their regulation in four other *osr* mutants (Excel file).
(0.09 MB XLS)

Table S5 Genes regulated in *dpy-9(e12)* mutants and their regulation in four other *osr* mutants (Excel file).
(0.59 MB XLS)

Table S6 Genes regulated in *dpy-10(e120)* mutants and their regulation in four other *osr* mutants (Excel file).
(0.70 MB XLS)

Table S7 Overlap between ORGs and genes regulated by eight different models of pathogen infection (Excel file).
(0.02 MB XLS)
